# Cluster of imported chikungunya virus infections in travelers returning from Seychelles to Berlin, December 2025–April 2026

**DOI:** 10.1016/j.nmni.2026.101812

**Published:** 2026-07-10

**Authors:** Gabriela Equihua Martinez, Andreas K. Lindner, Marlene Thielecke, Janina Hammer, Sarah Kotsias-Konopelska, Victor Max Corman, Till Dominik Best, Christian Drosten, Beate Kampmann, Paul Pitzinger

**Affiliations:** a Charité – Universitätsmedizin Berlin, Corporate Member of Freie Universität Berlin and Humboldt-Universität zu Berlin, Charité Center for Global Health, Institute of International Health, Berlin, Germany; b Charité – Universitätsmedizin Berlin, Corporate Member of Freie Universität Berlin and Humboldt-Universität zu Berlin, Institute of Virology, Berlin, Germany; c German Centre for Infection Research (DZIF), Associated Partner Site Charité, Berlin, Germany; d Labor Berlin-Charité Vivantes, Berlin, Germany

**Keywords:** Seychelles, Chikungunya, Outbreak, Arbovirus, Returning travelers

## Abstract

**Background:**

Mosquito-borne chikungunya virus (CHIKV) causes acute febrile illness with potentially persisting polyarthralgia, and its global resurgence in international travelers poses a risk of importation into non-endemic regions.

**Methods:**

We describe a cluster of ten travelers returning from the Seychelles to Berlin, Germany, between December 2025 and April 2026, diagnosed with acute CHIKV infection at an outpatient travel clinic. The diagnosis was confirmed by either reverse transcription polymerase chain reaction (RT-qPCR) or serological detection of Immunoglobulin M (IgM). Samples with a sufficient viral load were additionally sequenced.

**Results:**

Symptomatic patients (six male/four female; age range 27–64 years) presented 1–54 days after symptom onset; most reported fever and arthralgia at disease onset. CHIKV infection was confirmed by PCR in five of seven tested patients and by IgM serology in seven patients, including three PCR-positive cases who were initially seronegative early in infection. No dengue infections were detected. All patients were unvaccinated against CHIKV, and only one had sought pre-travel medical advice. Epidemiological assessment suggested multiple probable exposure sites within the Seychelles. Genomic sequencing was successfully performed in two samples.

**Conclusions:**

This cluster reflects ongoing CHIKV activity in the Seychelles and underscores the risk of international spread to Europe. Early diagnostic testing, particularly PCR during the acute phase, is essential to detect potential viremic patients. Improved pre-travel counseling, vaccination uptake, and clinician awareness are needed to reduce missed diagnoses and mitigate the risk of autochthonous transmission in regions with established *Aedes mosquito* populations.

## Highlights

• Ongoing chikungunya viral activity continues on different islands of the Seychelles with 10 infected travellers returning to Berlin
• Three patients were seronegative at the time of presentation, with infection detectable only by PCR.
• None of the patients were vaccinated against chikungunya and only one patient attended a pre-travel consultation.
• Clinicians should be aware of the emergence of arboviral diseases and consider PCR testing in acute cases.
• Persistent arthralgia, lasting longer than three months, was reported by 5/10 (50%) of the infected travellers

## 1 Introduction

Chikungunya virus (CHIKV) is a mosquito-borne arbovirus of global public health importance. It causes an acute febrile illness that is often accompanied by debilitating and persistent polyarthralgia. CHIKV belongs to the Alphavirus genus and is primarily transmitted by *Aedes aegypti*. The species *Aedes albopictus* serves as a secondary vector and is of increasing relevance in parts of Europe where it has established populations [1]. CHIKV was first described by Robinson in 1955 following an epidemic in the Southern Province of Tanganyika Territory, now known as Tanzania [2]. Since the early 2000s, research on CHIKV has expanded substantially following major outbreaks, progressing from limited epidemiological characterization [3] to more detailed molecular and translational studies [4]. CHIKV has undergone a global resurgence characterized by high incidence across endemic regions**,** while autochthonous clusters occur sporadically in countries in southern Europe, such as France and Italy [5]. Since the emergence of the E1-A226V mutation, CHIKV has shown adaptations that are thought to have increased its transmission efficiency, particularly via *Aedes albopictus* [6]. International travel could therefore facilitate the introduction of the virus into previously non-endemic regions via viremic individuals.

## 2 Methods

We conducted a retrospective observational case series due to a surge of CHIKV positive travelers returning from the Seychelles, who were diagnosed at the Institute of International Health at Charité - Universitätsmedizin Berlin, Germany, between December 2025 and April 2026. Returning travelers from the Seychelles with suspicion of arboviral infection prompted CHIKV testing and all positive cases were included. A positive case was defined either by detection of CHIKV-specific IgM, with or without Immunoglobulin (Ig)G antibodies by enzyme-linked immunosorbent assay (ELISA, Euroimmun) and/or a positive real-time reverse transcription polymerase chain reaction (RT-qPCR) for CHIKV. The data were extracted from medical records and evaluated by a team of trained physicians.

Initial laboratory testing for all suspected CHIKV cases included measurement of IgG and IgM antibodies against CHIKV and dengue virus (DENV). For patients presenting within ten days of onset of symptoms, RT-qPCR for CHIKV and antigen testing for DENV (SD BIOLINE Dengue Duo NS1 + Ab Combo Test™ Abbot) were performed on blood samples, taken on the same day. In two patients, RT-qPCR was additionally performed on serum samples collected >10 days after symptom onset despite an already established diagnosis based on CHIKV IgM positivity, as part of the extended laboratory work-up of this case series.

For complete CHIKV genome sequencing, the two samples with the highest viral loads, as indicated by low Ct values, were selected for a high-throughput RNA sequencing workflow as described previously [7]. The consensus sequences from the patient samples were generated and manually checked in Geneious Prime (Biomatters, Auckland, New Zealand) and subsequently compared with sequences in the NCBI GenBank database using BLAST.

## 3 Results

**Fig. 1:**
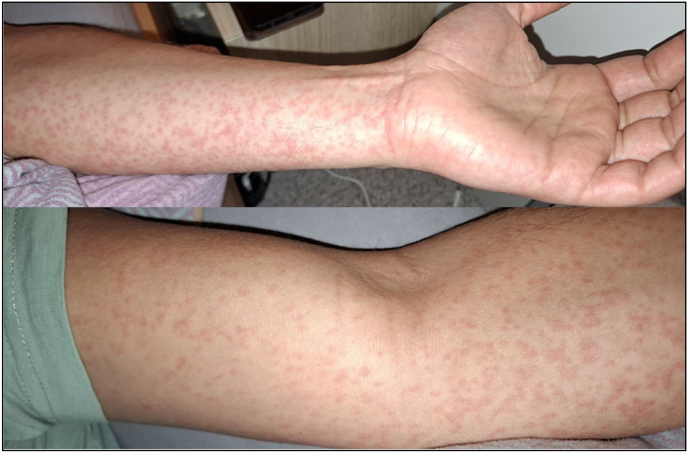
Diffuse erythematous maculopapular rash on the arms with extension to the palmar surface, with scattered areas of spared skin in a patient with chikungunya infection.

Ten returning travelers (six male and four female; age range: 27-64 years) from the Seychelles were diagnosed with CHIKV infection between December 2025 and April 2026 at the outpatient clinic in Berlin, Germany. Patients presented between 1 and 54 days after onset of symptoms (Table 1). All patients reported arthralgia, nine patients reported additionally fever and rash (Fig. 1) as main symptoms (Table 2), while prolonged arthralgia was the most frequently reported symptom during the subacute phase (9/10, 90%). All patients were first-time visitors to the Seychelles traveling for tourism independently, with a length of stay ranging from 7 to 18 days (Table 1). Notably, only one individual had sought a pre-travel medical consultation; none were vaccinated against CHIKV or were aware of the vaccine's availability. Based on assumed incubation periods in relation to exposure time, Praslin Island, Mahé (Misère), Silhouette Island, and La Digue were the most probable sites of infection; however, attribution remains uncertain because several patients travelled across multiple islands during the presumed exposure window, precluding definitive assignment to a single location.

**Table 1:** Characteristics from returning travelers diagnosed with CHIKV in from Seychelles to Berlin, Germany, December 2025 to April 2026.

Case	Sex	Age (in years)	Month of exposure	Length of trip (in days)	Onset of symptoms	Time of consultation	Probable Site of infection	Diagnostics		Pre-travel consultation
								Serology	RT-PCR	
1	Female	>60	Nov 2025	14	02.12.2025	09.12.2025	Praslin Island	IgG negative, IgM positive	Positive	No
2	Male	40-60	Dec 2025	14	22.12.2025	23.12.2025	La Digue	IgG negative, IgM negative	Positive	No
3	Male	20-40	Dec 2025	17	31.12.2025	14.01.2026	Mahé	IgG positive, IgM positive	Negative	No
4	Female	20-40	Dec 2025	17	25.12.2025	16.01.2026	Mahé	IgG positive, IgM positive	Negative	No
5	Male	40-60	Jan 2026	7	18.01.2026	26.01.2026	Praslin Island	IgG negative, IgM positive	Positive	No
6	Male	40-60	Feb 2026	18	18.02.2026	19.02.2026	Mahé	IgG negative, IgM negative	Positive	No
7	Male	40-60	Jan 2026	9	27.01.2026	25.02.2026	Silhouette Island	IgG positive, IgM positive	ND	No
8	Female	20-40	Jan 2026	9	22.01.2026	04.03.2026	Silhouette Island	IgG positive, IgM positive	ND	No
9	Female	40-60	Jan 2026	16	17.01.2026	12.03.2026	Praslin Island	IgG positive, IgM positive	ND	Yes
10	Male	20-40	Apr 2026	9	14.04.2026	16.04.2026	Mahé	IgG negative, IgM negative	Positive	No

Mahé: southernmost main island; Praslin Island: easternmost main island; Silhouette Island: westernmost main island, ND: not done.

**Table 2:** Most common reported symptoms by patients with CHIKV in the cohort, Berlin, Germany, December 2025 to April 2026.

Reported symptoms at diagnosis	Percentage of travelers (N = 10)	
	n	%
• Arthralgia	10	100
• Fever	9	90
• Exanthema/Rash	9	90
• Cephalgia	7	70
• Pruritus	5	50
• Swelling of joints	3	30
• Diarrhea	1	10
• Vomiting	1	10

### 3.1 Laboratory findings

Acute CHIKV infection was serologically confirmed in seven patients. Five of these seven patients also tested IgG-positive. Among the seven patients who underwent PCR testing, CHIKV-RNA was detected in five, including three individuals who were seronegative, showing no IgG and IgM. In these three cases, samples had been collected one day and two days respectively after onset of symptoms. DENV test results were negative. Zika virus serology was additionally performed in seven cases and was negative in all. All cases were notified to the Berlin public health authorities in accordance with mandatory reporting.

### 3.2 Genomic sequencing

The complete coding region of CHIKV was successfully sequenced from serum samples obtained from two patients in December 2025 and February 2026. Both sequences were assigned to the East/Central/South African genotype and were highly similar, differing by only six nucleotides across the coding region. Comparison with available genome sequences in the NCBI GenBank database identified the closest related sequences as those obtained from human samples collected in Réunion [8] between February and April 2025 (GenBank accession numbers PX799291-PX799293 and PV685653) [3]. These sequences differed by 2-5 nucleotides from the two Seychelles sequences, with all differences located within the coding region of the non-structural polyprotein.

### 3.3 Follow up investigation

Among the ten patients diagnosed with chikungunya, nine were successfully reached for follow-up. Persistent arthralgia lasting longer than three months, consistent with post-chikungunya chronic arthralgia, was reported by five patients (three females, two males). Symptoms ranged from mild to severe and predominantly involved the interphalangeal joints, but also affected larger joints and the axial skeleton. One woman had acquired CHIKV during pregnancy and reported persistent pain involving the hands, feet, spine, left knee and coccyx; she sought physiotherapy and osteopathic treatment for symptom relief. No pregnancy-related complications were reported. In four patients, symptom burden was substantial enough to warrant rheumatologic evaluation because of marked impairment in quality of life, affecting work, sports and daily activities. Analgesic therapy was modified in these cases, including the use of COX inhibitors, and two patients additionally received corticosteroids as part of their treatment regimen.

## 4 Discussion

Here we describe a cluster of CHIKV infected returning travelers from the Seychelles presenting at the outpatient clinic for tropical diseases in Berlin between December 2025 and April 2026. Autochthonous transmission of CHIKV has been documented in over 119 countries [5], encompassing approximately three-quarters of the global population [9]. Following two decades of relative quiescence, a renewed surge in CHIKV activity has been observed since 2024/25, with ongoing transmission documented through traveler-based surveillance systems [10]. Regions heavily impacted to date include several South American countries, India, Sri Lanka, and China's Guangdong province, as well as numerous nations and island territories bordering the Indian Ocean [11]. Among the latter, a CHIKV outbreak in Réunion, with 54,000 confirmed autochthonous cases, is currently declining [12].

Genomic analysis of two samples from the patient group suggests genetic similarity between the sequences from the Seychelles and those associated with the outbreak on Réunion in 2025. However, the currently available sequence data, particularly also from other locations in this geographic region, are insufficient to establish a direct link between the two outbreaks.

Between December 2024 to December 2025, more than 115,000 tourists visited the Seychelles including over 82,000 from Europe [13]. In February and March 2026, CDC and ECDC issued CHIKV outbreak notifications for the Seychelles [14], alerting of potential infections in returning travelers to Europe and other regions.

A first larger autochthonous CHIKV outbreak in Europe already occurred in Italy in 2007 [15], followed by subsequent local transmission clusters in France and Italy [16]. After an outbreak in Réunion by mid-July 2025, more than 1900 CHIKV cases had been recorded in mainland France alone, with the northernmost cases in the Alsace region bordering Germany [17]. Viremic travelers returning from the Seychelles and other areas experiencing ongoing CHIKV epidemics may facilitate the introduction of CHIKV into areas with competent vectors and contribute to future autochthonous transmission during the vector-active season. Big data–based surveillance methods, such as digital health data, travel analytics, and environmental modelling, remain largely underutilized despite their potential to improve the monitoring of chikungunya transmission and outbreak detection [18]. Projections suggest that climatic suitability in Europe for *Aedes albopictus* will persist and likely expand [19]. The containment of *Aedes* breeding sites should therefore consequently be reinforced in Europe ahead of the warmer spring and summer months [20]. Established presence and northward spread of *Aedes albopictus* across Europe elevates the risk of autochthonous arboviral transmission, including CHIKV [21]. Although the patients in this series returned during winter and early spring, when *Aedes albopictus* activity in Germany is expected to be minimal and the immediate risk of local transmission is therefore low, repeated importation and transit of viremic cases to countries with more favorable climate conditions remain relevant. In this context, populations in these locations are largely immunologically naïve to CHIKV and disproportionately elderly, which increases the risk of rapid outbreak escalation and severe disease [22]. Recommendations for personal protective measures, such as repellents against day-active mosquitoes, should be guided by local entomological assessments and vector density.

Chikungunya fever remains rare in Europe. Clinical manifestations in patients may be unfamiliar to clinicians, which can delay timely diagnosis [23]. Prolonged arthralgias after fever episodes during or after travel in endemic regions should trigger testing for CHIKV. A rise in mortality in regions affected during previous outbreaks [24] and although rare, cases of severe disease in otherwise healthy young patients have been reported [25]. Aging and associated comorbidities in European populations increase the risk of severe disease, hospitalization, mortality, and chronic manifestations of CHIKV infection [26]. Within this cluster, one patient was over 60 years of age, and another had a pre-existing neurological disorder, placing them in the high-risk category. Although our observations are based on a small cohort, the high proportion of patients who reported persistent arthralgia highlights that chikungunya should not be regarded solely as an acute self-limiting illness. Instead, it may result in chronic musculoskeletal morbidity with a substantial impact on quality of life, emphasizing the importance of long-term follow-up and, in some cases, prolonged multidisciplinary management.

For three patients, a prompt definitive diagnosis was only possible by RT-qPCR while serology was negative at presentation. PCR testing may not be readily available in all health care settings; therefore, diagnostic delays can result in unnecessary costs from repeated tests, inappropriate treatments, and additional clinic visits [27]. Critically, a missed or delayed diagnosis also heightens the risk of onward transmission and potential outbreaks in areas with competent mosquito vectors. Thus, clinical vigilance is advised in travelers returning from Indian Ocean islands, particularly the Seychelles, who present with unexplained fever, arthralgia, or other musculoskeletal symptoms. In the early acute phase, PCR should be prioritized over serology.

These observations, namely that none of the patients had been vaccinated against CHIKV, that most were unaware of the vaccine's availability, and that only one had attended a pre-travel medical consultation, indicate several missed opportunities for prevention. Increasing awareness of CHIKV vaccination and pre-travel risk assessment among both travelers and healthcare providers is therefore essential to improve vaccine uptake and reduce the risk of virus importation.

Until recently, individual level prevention relied exclusively on mosquito avoidance and vector control. Two vaccines are now licensed in the European Union: the live attenuated vaccine Ixchiq® and the non-replicating virus like particle (VLP) vaccine Vimkunya® [[28], [29], [30]]. Both are administered as a single intramuscular dose and have demonstrated high seroprotection rates in phase 3 trials across adult age groups, with predefined neutralizing antibody thresholds used as surrogate correlates of protection in the absence of clinical endpoint data [30].

Post marketing safety data for Ixchiq® have shown rare but serious adverse events, occurring in older adults with multimorbidity and often presenting as systemic chikungunya like reactions or neurological events [28,30]. These signals led to a formal EU pharmacovigilance referral, resulting in a comprehensive EMA safety review and strengthened product information, while the indication for use in individuals aged ≥12 years was maintained, and routine pharmacovigilance was intensified [28]. In contrast, clinical trial data and early post authorization experience suggest a more favorable reactogenicity and safety profile for Vimkunya®, including in adults ≥65 years [29,30].

Against this background, the German Standing Committee on Vaccination (Ständige Impfkommission, STIKO) recommends CHIKV vaccination for travelers to areas with ongoing outbreaks or repeated or long term stays in endemic regions, particularly in individuals with an increased risk of chronic or severe disease, as well as for selected occupationally exposed groups [29]. In accordance with these recommendations and the available immunogenicity and safety data, Ixchiq® is recommended for immunocompetent persons aged 12–59 years, whereas Vimkunya® can additionally be recommended from 60 years of age and is thus the preferred option in older or multimorbid adults [[28], [29], [30]].

Regardless of vaccine availability, consistent mosquito-bite prevention remains the cornerstone of protection against arboviral infections. Repellents are widely accessible, including to travelers who do not seek pre-travel medical advice. Public health campaigns, travel information, and travel-related apps should therefore promote their regular use, particularly during periods of *Aedes* activity. Similar advice should also be provided to recently CHIKV-diagnosed travelers in their home countries to reduce the risk of onward transmission to locally established mosquito vectors.

As most patients in our cohort had not sought pre-travel medical advice, additional communication channels are needed to reach travelers. Timely public health alerts, digital travel platforms, social media platforms, airlines, pharmacies, and travel providers could improve awareness of ongoing arboviral outbreaks and reinforce recommendations for mosquito-bite prevention and vaccination where appropriate.

Presently there are no approved antiviral therapies to treat CHIKV, but there are currently endeavors underway in development of antiviral drugs acting selectively on nonstructural proteins of CHIKV [31] and although in early stage may render promising in the coming years [32]. Treatment guidelines vary but focus on supportive care, including pain and fever management with analgesics, adequate hydration, and monitoring for complications [33]; in cases of persistent arthralgias, rheumatological assessment may be warranted [34].

## 5 Conclusion

This patient cluster highlights the evolving hazard posed by regional arboviral outbreaks and the diagnostic challenges they present in non-endemic settings. To prevent missed diagnoses during the early acute phase, PCR testing should be considered. Our findings suggest that CHIKV awareness among travelers remains limited. The high proportion of patients with persistent arthralgia in our cohort further emphasizes the considerable long-term burden of chikungunya and the importance of recognizing its potential for progression to chronic disease. Healthcare professionals should emphasize rigorous mosquito protection and prioritize integrating real-time global epidemiological data into routine clinical practice, which is essential for the timely identification of imported cases and the mitigation of autochthonous transmission risks within Europe.

## Ethical statement

No ethics committee approval was required for this observational study. All data were anonymized in accordance with relevant legal and ethical standards. Written informed consent for publication was obtained from all patients.

## Declaration of generative AI and AI-assisted technologies in the writing process

During manuscript preparation, the authors used ChatGPT 5.4 (OpenAI) to support language editing and improve readability. The tool was not permitted to introduce content from external sources. All outputs were reviewed and edited by the authors, who take full responsibility for the final content.

## Funding statement

This work was partially supported by the EU4Health Programme (grant no. 101102733; DURABLE; to C. Drosten).

## CRediT authorship contribution statement

**Gabriela Equihua Martinez:** Conceptualization, Data curation, Formal analysis, Funding acquisition, Investigation, Methodology, Project administration, Resources, Supervision, Validation, Visualization, Writing – original draft, Writing – review & editing. **Andreas K. Lindner:** Formal analysis, Methodology, Supervision, Visualization, Writing – original draft, Writing – review & editing. **Marlene Thielecke:** Investigation, Methodology, Visualization, Writing – original draft, Writing – review & editing. **Janina Hammer:** Investigation, Methodology, Visualization, Writing – original draft, Writing – review & editing. **Sarah Kotsias-Konopelska:** Investigation, Methodology, Visualization, Writing – original draft, Writing – review & editing. **Victor Max Corman:** Conceptualization, Data curation, Formal analysis, Investigation, Methodology, Validation, Visualization, Writing – original draft, Writing – review & editing. **Till Dominik Best:** Conceptualization, Data curation, Formal analysis, Investigation, Methodology, Validation, Visualization, Writing – original draft, Writing – review & editing. **Christian Drosten:** Formal analysis, Funding acquisition, Investigation, Methodology, Supervision, Validation, Writing – review & editing. **Beate Kampmann:** Formal analysis, Methodology, Supervision, Validation, Writing – review & editing. **Paul Pitzinger:** Conceptualization, Data curation, Formal analysis, Investigation, Methodology, Project administration, Resources, Supervision, Validation, Visualization, Writing – original draft, Writing – review & editing.

## Declaration of competing interest

The authors declare that they have no known competing financial interests or personal relationships that could have appeared to influence the work reported in this paper.

## Data Availability

Sequence data will be made publicly available in the NCBI GenBank database, with accession numbers to be provided upon release. Only data necessary for cluster description are included in this report. Individual case-level data are not available due to data protection and privacy regulations.
